# Induction of Apoptosis in Human Leukemia Cell Line (HL60) by Animal’s Venom Derived Peptides (ICD-85)

**Published:** 2012

**Authors:** Abbas Zare Mirakabadi, Zahra Shahramyar, Hasan Morovvati, Mohsen Lotfi, Ali Nouri

**Affiliations:** a *Department of Venomous Animals and Antivenom Production, Razi Vaccine and Serum Research Institute, Karaj, Iran.*; b *Quality Control Department, Razi Vaccine and Serum Research Institute, Karaj, Iran.*; c *Electron Microscopy Department, Razi Vaccine and Serum Research Institute, Karaj, Iran. *; d *Cancer Research Institute, Tehran University of Medical Science, Tehran, Iran.*

**Keywords:** Anticancer, Apoptosis, HL60, ICD-85, Venom, DNA fragmentation, Cytotoxicity

## Abstract

Our previous studies revealed an inhibitory effect of ICD-85 (Venom derived peptides) on breast cancer cell line MDA-MB231. ICD-85 was also confirmed by *in-vivo *studies to suppress the breast tumor in mice. However, the exact mechanism of ICD-85 was unknown. Hence, the present study was undertaken to assess the mechanism of ICD-85 effect as an anti-proliferative agent of cancer cells. The effect of ICD-85 on proliferation of HL-60 cancer cells was determined by using the MTT assay. The morphological changes of ICD-85 treated HL-60 cells were observed under transmission electron microscope (TEM). DNA fragmentation analysis was also carried out using gel electrophoresis. ICD-85 induced the marked inhibition of HL60 cell proliferation with an IC_50_-value of 0.04 μg/mL following 24 h of incubation. ICD-85 treated cells when compared with untreated cells, showed nuclear material condensation, endoplasmic reticulum dilation, mitochondria swelling or degradation, increased cytoplasmic vacuoles, reduction or disappearance in cytoplasmic process and decreased nuclear/cytoplasmic ratio was observed. The characteristic DNA ladder formation of ICD-85-treated cells in agarose gel electrophoresis confirmed the results obtained through the electron microscopy. The results of the present study indicated that ICD-85 inhibited the cancer cell proliferation by inducing cell apoptosis.

## Introduction

Venom of some animals such as snake and scorpion had been reported to be cytotoxic on tumor cells which were mediated through inducing apoptosis in the target cells (1-3). There are some reports that present the cytotoxic activity of various snake venoms, *in-vivo *and *in-vitro*, by employing melanoma and chondrosarcoma cells (4). A tumor is a disease characterized by uncontrolled proliferation and absence of apoptosis. Apoptosis or programmed cell death is a fundamental event that plays an important role in the homeostasis and development of an organism (5-8).

The potential mechanism for a cell to undergo apoptosis exists in a balance between its induction and inhibition factors (9). Venom of some animals such as snakes and scorpions had been reported to be cytotoxic on tumor cells which were mediated through inducing apoptosis in the target cells (1, 3, 4). Lyons *et al. *reported Chlorotoxin, a scorpion derived peptide, specifically binds to gliomas and tumors of neuroectodermal origin (10). Report of clinical trial of marine derived anti-cancer peptide also showed thesignificant role of natural peptides that would be considered as future hope in cancer treatment (11). Silva *et al. *evaluated the action of the venoms from *Crotalus durissusterrificus *and *Bothrops jararaca *on Ehrlich ascites tumors and found that both venoms act directly on tumor cells. They further postulated the various mechanisms involved in the anti-tumor activity (12).

Our previous studies proved that ICD-85 (Venom derived peptides) can inhibit the growth of various cancer cell lines including HeLa, Vero and MDA-MB231 (13). Our *in-vivo *studies on naturally developed breast tumor in mice also showed the suppressive effect of ICD-85 on tumor growth (14). However, the mechanism of inhibition was unknown. The present study was undertaken to determine the mechanism by which ICD-85 act as inhibitory agent for cancer cells.

## Experimental

*Venom derived peptide (ICD-85)*

The active fraction of ICD-85 is a combination of three peptides, ranging from 10,000 to 30,000 Da, derived from the venoms of an Iranian brown snake (*Agkistrodon halys*) and a yellow scorpion (*Hemiscorpius lepturus*). This fraction was formulated and provided by the corresponding author. The ICD-85 peptides were selected based on a study of crude venom cytotoxicity. The crude venom showed antigrowth activity on the MBA-MD231 cell line. Then, the venoms were fractionated; the active peptides were isolated and, subsequentially, tested on the same cell line (13).

*Assessment of cell viability and proliferation using MTT assay*

Cell proliferation and cytotoxicity were determined using the MTT chromophore (Sigma-Aldrich) to estimate cell number spectrophotometrically as a function of the oxidative status of the cells. The tetrazolium salt MTT used to measure the metabolic activity of viable cells. Tetrazolium salts are reduced to formazan by mitochondrial succinate dehydrogenase, an enzyme which is only active in cells with an intact metabolism and respiratory chain. The formazan is quantified with a spectrophotometer and correlates with the number of viable cells. Briefly, tissue culture plates containing 100 μL of a cell suspension (250000, cells/mL) were incubated with 100 μL of various ICD-85 concentrations from 4 × 10^-15^ to 4 × 10 μg/mL. After incubation for 24 h, 10 μL/well of the labeling mixture, consisting of 5 mg/mL MTT in phosphate-buffered saline solution (PBS), pH 7.2 were added to each well and incubation was continued for 4 h in the dark and then the culture medium was removed and 100 μL of isopropranol and HCL, 1N were added to each well to solubilize the formazan formed. The plate was shaken gently for 10 min. and the absorbance (OD) at 570 nm was recorded using a microplate reader. A blank well containing only culture medium and the labeling mixture was included for background correction (15).

*Determination of IC*_50_ *values*

The concentrations that decreased 50% of cell proliferation is named Inhibitory Concentration 50% (IC_50_) which were determined using serial dilutions of ICD-85 (typically 4 × 10^-15^ to 40 μg/mL). Control cells were treated with phosphate buffer without ICD-85.

The viability calculation was determined as below:

(%)Viability = (OD of treated cells / OD of untreated cells (control)) × 100

%Inhibition = 100 - %viability

Here, IC_50_ was taken as the concentration that caused 50% inhibition of the cell viability and was calculated through the logit method (16).

*Electron microscopy*

Cell suspensions were incubated with 100 μL 0.04 μg of ICD-85 for 24 h and were pelleted by centrifugation. Samples were routinely processed through fixation with 2.5% glutaraldehyde in 0.1 M phosphate buffer, with pH of 7.2 for 2 h, and post fixed with 1% Osmium tetroxide for 1 h. Then, after dehydration in graded series of ethanol, it was embedded in epoxy 812 resin and sectioned for TEM. Finally, uranyl acetate and lead citrate stained sections were examined using electron microscope of Phillips-400 with an accelerating potential of 80 KV.

*Release of lactate dehydrogenase (LDH)*

Lactate dehydrogenase (LDH), which is a soluble cytosolic enzyme present in most eukaryotic cells, releases into culture medium upon cell death due to the damage of plasma membrane. The increase of the LDH activity in culture supernatant is proportional to the number of lysed cells. LDH catalyses the reduction of NAD+ to NADH in the presence of L-lactate, while the formation of NADH can be measured in a coupled reaction in which the tetrazolium salt INT is reduced to a red formazan product. The amount of the highly colored and soluble formazan can be measured at 490 nm spectrophotometrically. A quantitative cytotoxicity assay based on the release of lactate dehydrogenase (LDH; EC 1.1.1.27) was performed 24 h after exposure to various ICD-85 concentrations *i.e*. from 4 × 10 to 4 × 10^-5^μg/mL (17).

*DNA fragmentation assay*

For DNA fragmentation detection, HL60 cells (10^6^cells/mL) treated with ICD-85 of concentration 4 and 0.04 μg/mL for 24 h. and then were centrifuged at 3000 rpm for 5 min. Cells were washed twice with PBS, pelleted, and incubated in 200 μL of lysis buffer (10 mM Tris–HCl and 10 mM EDTA (pH 8.0), 5% SDS, and 1 mg/mL proteinase K) for 1 h at 55°C and treated with 0.5 mg/mL RNase. DNA was extracted with phenol/chloroform/isoamyl alcohol (25:24:1 v/v ) and precipitated with 100 μL ammonium acetate of 7.5 M and 3 volumes of ethanol. DNA fragmentation analyzed by electrophoresis for 1 h at 75 V on a 1% agarose gel containing ethidium bromide, then observed and photographed under UV light (15).

*Statistical analysis*

Data were analyzed using student’s t-test with statistical significance for p < 0.05.

## Results and Discussion

*Lactate dehydrogenase (LDH)*

The mean activity of LDH enzyme in control sample (untreated HL60 cells) was found to be 52 ± 3.50 U/L. LDH determination of cultured media of HL60 cells exposed to various concentration of ICD-85 revealed that no significant change occurred as compared with untreated cells. 

**Figure 1:**
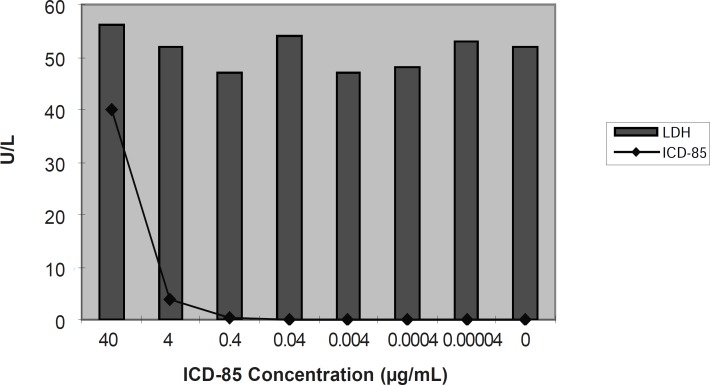
Extracellular release of LDH in cultured media of HL60 cells exposed to ICD-85. release of lactate dehydrogenase was performed 24 h after exposure to various ICD-85 concentrations from 4 ×10 to 4×10^-5^μg/mL (17).

*Effect of ICD-85 on cell proliferation*

**Figure 2:**
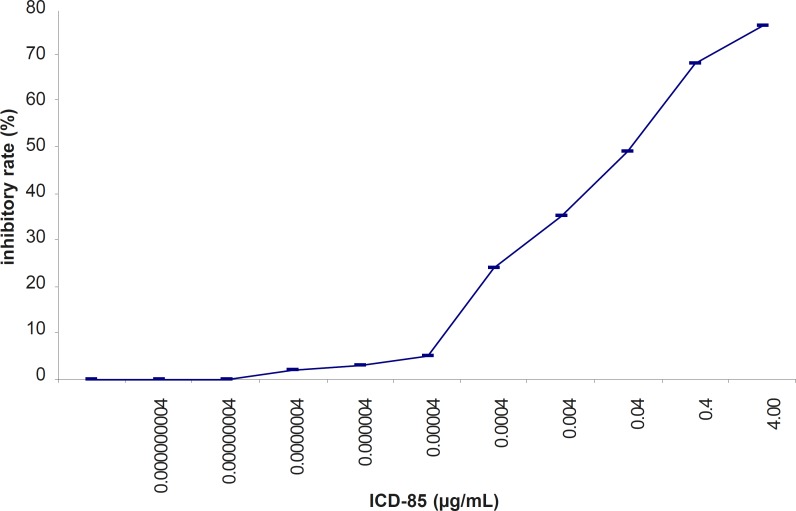
Inhibitory effect of ICD-85 on the viability of HL-60 cells treated with various concentration of ICD-85 for 24 h. MTT assay was used to detect the cell viability

The inhibitory effect of ICD-85 on the proliferation of HL-60 cancer cells was determined by using the MTT assay. ICD-85 inhibited the growth of HL-60 cancer cells in a concentration-dependent manner. The IC50 value of ICD-85 was 0.04 ± 0.015 μg/mL after 24 h incubation (Figure 2).

*Effect of ICD-85 on the morphology of cells*

**Figure 3:**
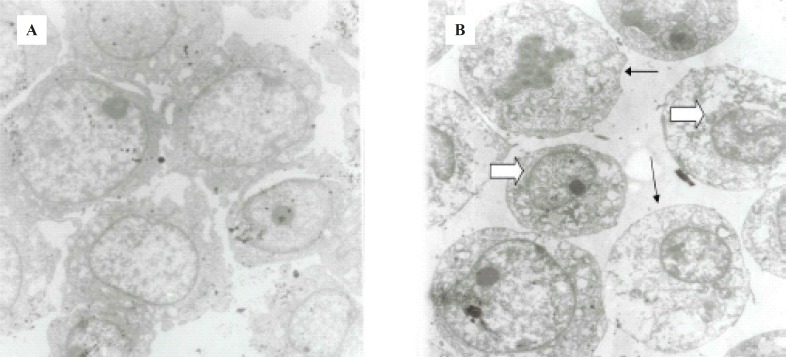
(a) Untreated HL60 cells. (b) 24 h. ICD-85 treated HL60 cells. Cell sections marked change in nuclear/cytoplasmic ratio, condensing nuclear material peripherally (large arrow), increased cytoplasmic vacuoles, and reduction or disappearance in cytoplasmic process (small arrows) was seen in comparison in ICD-85 treated cells. Magnification (4800 X).

Morphological studies of ICD-85-treated as well as untreated HL-60 cells were carried out by TEM. Uniform cells with normal morphology were seen in the control group (Figure 3 (a)), whereas ICD-85 treated cells (Figure 3 (b)), showed marked shrinkage and change in nuclear/cytoplasmic ratio in favored of cytoplasm which was observed to be due to nuclear material condensation,endoplasmic reticulum dilation, mitochondria swelling or degradation, increased cytoplasmic vacuoles and reduction or disappearance in cytoplasmic process. These results suggest that ICD-85 was able to induce alterations in morphological features of HL-60 cells in electron microscopy level of studies.

*Effect of ICD-85 on DNA fragmentation*

**Figure 4:**
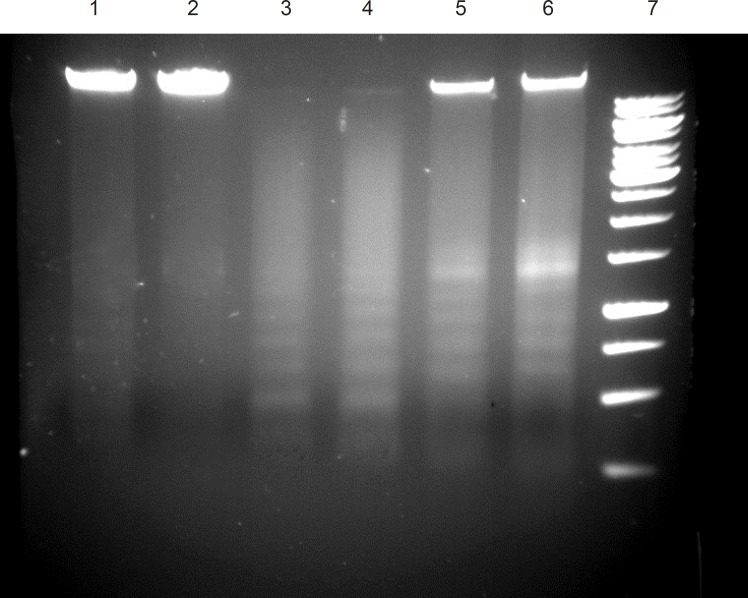
DNA fragmentation in ICD-85 treated cells. untreated cells (Lane 1, 2), cells treated with 8 μg/mL camptothecin (Lane 3 ), cells treated with 4 μg/mL camptothecin (Lane 4), cells treated with 0.04 μg/mL ICD-85 (Lane 5), cells treated with 4 μg/mL ICD-85 (Lane 6), marker 1Kb (lane 7).

DNA was isolated from the HL-60 cancer cells cultured in the presence of ICD-85 at various concentrations for 24 h. The characteristic partial ‘ladder’ pattern (lanes 5 and 6) of apoptosis was observed at 0.04 and 4 μg/mL of ICD-85. A full DNA ladder is visible (lanes 3 and 4), when isolated DNA of 2 × 10^6^ HL-60 cells treated with 4 and 8 μg/mL camptothecin as positive control for 24 h (Figure 4).

## Discussion

Our previous studies proved that ICD-85 (Venom derived peptides) can inhibit the growth of various cancer cell lines including HeLa, Vero and MDA-MB231 (13). Our *in- vivo *studies on naturally developed breast tumor in mice also showed the suppressive effect of ICD-85 on tumor growth (14). To study the mechanism of growth inhibition of ICD-85 on cancer cells, HL60 cell line was used, because it is a well established fact that this cell line is a practical model for studying the induction of apoptosis by antitumor agents (18-20). In the present work, MTT assay confirmed the cytotoxic nature of ICD-85 and when IC_50_ of this compound was determined it was found to be 0.04 μg/mL. The concentration of 0.04 μg/mL as IC_50 _of ICD-85 is an indicator of high potency of these peptides as antiprolifrative agent on HL60 cells. However as shown in the Figure 4 complete apoptosis of cells was not observed. This may be due to the time limitation. Lactate dehydrogenase determination in cultured media of HL60 cells, treated with various concentrations of ICD-85 showed no significant rise as compared to untreated cells (Figure 1). The LDH leakage assay is based on the release of the enzyme into the culture medium after cell membrane damage while the MTT assay is mainly based on the enzymatic conversion of MTT in the mitochondria (21). Kathikeyan et al assessed the cytotoxic effect of snake venom by the release of LDH from HeLa and Hep2 cells after treating with the venom which exhibited positive results with a reduced amount of LDH. Reduced LDH release showed cell recover to normal function and venom has inhibition mechanism against the cancer cells (22). At the present study transmission electron microscopic of the HL60 cells exposed to ICD-85 revealed a sort of shrinkage in the cells. Condensation of cell cytoplasm and chromatin as well as cell shrinkage in ICD-85 treated HL60 cells was also observed (Figure 3 b). These results are supported by our previous studies on MDA-MB231 cell line exposed to ICD-85 which showed the shrinkage of cells under light microscopy (13). Apoptosis is characterized by condensation of cell cytoplasm and chromatin, cell shrinkage, nuclear breakdown, DNA fragmentation and cell fragmentation into apoptotic bodies (23). On the other hand, necrosis is characterized by the swelling of the cells, formation of microvessicles, and leakage of the cytoplasm (24, 25). The characteristic ‹ladder› pattern of apoptosis was observed in isolated DNA of HL60 cells exposed to ICD-85. DNA laddering is one the most important indicator of apoptosis in cells (26). In this study Champtothecin was used as a positive control (27). The DNA laddering pattern of HL60 cells exposed to ICD-85 and champtothecin looks almost similar (Figure 4). The DNA laddering pattern of HL60 cells exposed to ICD-85 as compared to untreated cells also reveals the induction of apoptosis. Some published data indicate peptides composition in various venom of scorpion and snakes are able to induce apoptosis in cancer cells. For example Gupta et al showed that the Indian scorpion (*H. bengalensis*) venom possessed antiproliferative, cytotoxic and apoptogenic activity against human leukemia cells. In addition, under scanning electron microscope it was observed that the scorpion venom treated U937 and K562 cells showed a very high degree of membrane blebbing as compared to the control cells (28). Trummal K. *et al *demonstrated VLAIP from *V. lebetina *snake venom is a potent promoter of apoptosis in vascular endothelial cells (29). Karthikeyan et al showed the agarose gel electrophoresis of chromosomal DNA isolated from the EAC cells revealed the fragmented DNA of cells exposed to medium dose of snake (*Hydrophis spiralis*) venom (30).

Clinicians know that cytotoxic drugs kill cancer cells, but unfortunately, most of the currently used drugs are highly toxic to a diversity of normal tissues (31). For example, cisplatin has been shown to be one of the most reliable chemotherapy drugs for the treatment of a variety of cancer. However, its use has been limited because of its toxicity to non-malignant cells (32). On the other hand, venom of some animals such as snake and scorpion had been reported to be cytotoxic on tumor cells which were mediated through inducing apoptosis in the treated cells (1 & 3). These natural compounds may regulate tumorigenesis and/or the growth of cancer cells by inducing apoptosis in malignant cells. Honda et al demonstrated an inhibiting effect on tumor cell growth by arresting the cells to the G1-phase and decreasing the S-phase cells in leukemia (HL-60) (33). Apoptosis can be inducted in HL-60 cells by several agents such as camptothecin (Figure 4), etoposide, cisplatin and 5-azacytidine (34). The mechanisms underlying these effects vary depending on the beginning stimulus, but a common feature is the activation of endonucleotidases leading to DNA fragmentation (35, 36). Endonucleotidase activation may result from interruption of DNA supercoil structure interference with DNA repair mechanisms (37) or interference with normal cellular signaling pathways (Bergamaschi *et al*., 1993) (38). In conclusion, results of the present study based on DNA fragmentation, electron microscopy images, LDH and MTT assay suggest that ICD-85 has a significant antiprolifrative effect on HL-60 cells, through induction of apoptosis. Since the molecular weight of these peptides are higher than they would penetrate the cell membrane of HL60 cells, the apoptosis might be exerted via membrane receptors on cell surface. 
